# The presence of differentiated C2C12 muscle cells enhances toxin production and growth by *Clostridium perfringens* type A strain ATCC3624

**DOI:** 10.1080/21505594.2024.2388219

**Published:** 2024-08-27

**Authors:** Jihong Li, Sameera Sayeed, Bruce A. McClane

**Affiliations:** Department of Microbiology and Molecular Genetics, University of Pittsburgh School of Medicine, Pittsburgh, PA, USA

**Keywords:** *Clostridium perfringens*, alpha toxin, perfringolysin O, C2C12 muscle cells, bacterial growth, regulation of toxin production

## Abstract

*Clostridium perfringens* type A causes gas gangrene, which involves muscle infection. Both alpha toxin (PLC), encoded by the *plc* gene, and perfringolysin O (PFO), encoded by the *pfoA* gene, are important when type A strains cause gas gangrene in a mouse model. This study used the differentiated C2C12 muscle cell line to test the hypothesis that one or both of those toxins contributes to gas gangrene pathogenesis by releasing growth nutrients from muscle cells. RT-qPCR analyses showed that the presence of differentiated C2C12 cells induces *C. perfringens* type A strain ATCC3624 to upregulate *plc* and *pfoA* expression, as well as increase expression of several regulatory genes, including *virS/R*, *agrB/D*, and *eutV/W*. The VirS/R two component regulatory system (TCRS) and its coupled Agr-like quorum sensing system, along with the EutV/W TCRS (which regulates expression of genes involved in ethanolamine [EA] utilization), were shown to mediate the C2C12 cell-induced increase in *plc* and *pfoA* expression. EA was demonstrated to increase toxin gene expression. ATCC3624 growth increased in the presence of differentiated C2C12 muscle cells and this effect was shown to involve both PFO and PLC. Those membrane-active toxins were each cytotoxic for differentiated C2C12 cells, suggesting they support ATCC3624 growth by releasing nutrients from differentiated C2C12 cells. These findings support a model where, during gas gangrene, increased production of PFO and PLC in the presence of muscle cells causes more damage to those host cells, which release nutrients like EA that are then used to support *C. perfringens* growth in muscle.

## Introduction

*Clostridium perfringens* is a Gram-positive, spore-forming anaerobic bacterium that causes a broad range of human and animal diseases, e.g. gas gangrene, food poisoning, antibiotic-associated diarrhoea and enterotoxemia [1–4]. Toxins play a major role in the virulence of this bacterium, which produces an impressive armamentarium of >20 different toxins [1–3,5]. However, toxin production varies greatly between isolates, allowing classification of *C. perfringens* isolates into seven types (A-G) based upon their carriage of six toxin genes (*plc* or *cpa*, *cpb*, *etx*, *iota*, *cpe,* and *netB*) [6].

The only typing toxin gene carried by *C. perfringens* type A isolates is the *cpa* (also referred to as *plc*) gene encoding alpha toxin (CPA or PLC), which has phospholipase C and sphingomyelinase activities [3,6,7]. Although not used for toxin typing purposes, type A isolates also produce perfringolysin O (PFO), which is a pore-forming toxin [8]. *C. perfringens* type A is the leading cause of traumatic clostridial myonecrosis (gas gangrene), which develops following an injury that introduces type A bacteria deeply into soft tissue, including muscle [7,9,10]. *C. perfringens* then grows in the muscle and produces toxins and enzymes that cause a rapidly spreading tissue necrosis [7,9,10]. Both CPA and PFO contribute to gas gangrene [11]. Beyond causing localized tissue necrosis, these toxins eventually enter the circulation, which results in multiple organ failure [10,12,13]. Without effective treatment, *C. perfringens* myonecrosis is nearly always rapidly fatal [10].

CPA and PFO cause necrosis, inhibit the tissue inflammatory response, and affect vascular flow, effects that likely contribute to gas gangrene [3,7,9,12]. However, CPA and PFO may play additional roles in gas gangrene. It has long been proposed, but to our knowledge never experimentally shown that type A strains may also use CPA and PFO to obtain substances from host cells to use for their growth and metabolism. Obtaining growth and energy substrates from host cells should be particularly important for type A isolates during gas gangrene considering that nutrient acquisition from blood becomes difficult as gas gangrene is established and blood flow is reduced in necrotic tissue.

Consistent with the possibility that *C. perfringens* uses toxins to obtain growth substances from host cells, our lab previously demonstrated that *C. perfringens* type C strains (which cause enterocolitis and enterotoxemia) produce more toxins, including beta toxin (CPB), PLC, and PFO when cultured in the presence of human enterocyte-like Caco-2 cells [14]. Both the Agr-like quorum sensing system (Agr-like QSS) and the VirS/VirR two-component regulatory systems (VirS/R TCRS) can be involved in mediating this Caco-2 cell-induced increase in toxin production [14,15]. This regulatory linkage is consistent with, i) recent findings showing that VirS is a receptor for the signalling peptide (AgrD) of the *C. perfringens* Agr-like QSS [16] and ii) several labs having demonstrated that the Agr-like QSS and VirS/R TCRS control PLC, PFO, and CPB production [15,17–25]. VirR directly controls PFO and CPB production due to the presence of two VirR boxes upstream of the *pfoA* or *cpb* genes [26,27]. However, VirR boxes are not located upstream of the *plc* gene; instead, VirR indirectly regulates PLC production by controlling the expression of a small regulatory RNA named VR-RNA [23]. A recent study demonstrated that the Agr-like QSS system is important for gas gangrene induced by type A strain ATCC 3624 in a mouse model [28].

In addition to the VirS/R TCRS, the *C. perfringens* genome encodes over 20 other predicted TCRSs [29,30]. One of those is the EutV/EutW (EutV/W) TCRS, which is encoded in the *eut* region of the chromosome that contains 19 genes involved in processing, uptake, and metabolism of ethanolamine (EA) [31,32]. The *eutV/W* genes are expressed from their own promoter [32]. Phosphatidylethanolamine (PE) is an abundant phospholipid in both mammalian and bacterial cell membranes [33]. *C. perfringens* PLC can degrade PE to EA, which is known to increase transcription of *eut* utilization genes [31]. In addition, *C. perfringens* can utilize EA as an energy source for growth and studies using a *eutV* mutant of HN13, a type A strain 13 derivative, suggested that EA utilization promotes gas gangrene [31,32]. Control of expression of EA transport and utilization genes by regulators such as the VirS/R TCRS and *virX* is dependent on environmental conditions. For example, when neither glucose nor EA is available, the VirS/R TCRS induces expression of genes encoding proteins involved in EA transport and utilization [31,32].

Differentiated mouse C2C12 muscle cells are the most commonly used cellular model to mimic muscle *in vitro*, including studies of gas gangrene induced by *C. perfringens* type A or its toxins [34–38]. Therefore, the current study used the differentiated mouse muscle C2C12 cells as an *in vitro* model to study type A interactions with muscle cells during gas gangrene, with the goal of improving the understanding of *C. perfringens* gas gangrene pathogenesis. Among the important questions, this study sought to investigate were: Does the presence of differentiated C2C12 cells induce rapid upregulation of *plc* and *pfoA* expression by the type A strains that cause gas gangrene? If so, are the VirS/VirR TCRS and the Agr-like QSS important? In the presence of differentiated C2C12 cells, is the EutV/W TCRS involved in regulating toxin production? Can EA alone modulate toxin production? Most importantly, the present study sought to answer the fundamental, but unproven, question regarding whether *C. perfringens* produces toxins, at least in part, in order to generate host substances for its growth?

## Results

### The presence of differentiated mouse muscle C2C12 cells increases *plc* and *pfoA* toxin gene expression by *C.* *perfringens* type A strain ATCC3624

Since infection of Caco-2 cells with *C. perfringens* type C strains, which cause enteric disease, was shown to induce upregulation of expression of several toxin genes, including *plc* and *pfoA* [14], the current study used differentiated C2C12 mouse muscle cells to test whether a similar effect also occurs in a gas gangrene-relevant environment, i.e. incubation of a type A strain in the presence of differentiated C2C12 muscle cell line.

**Figure 1.**
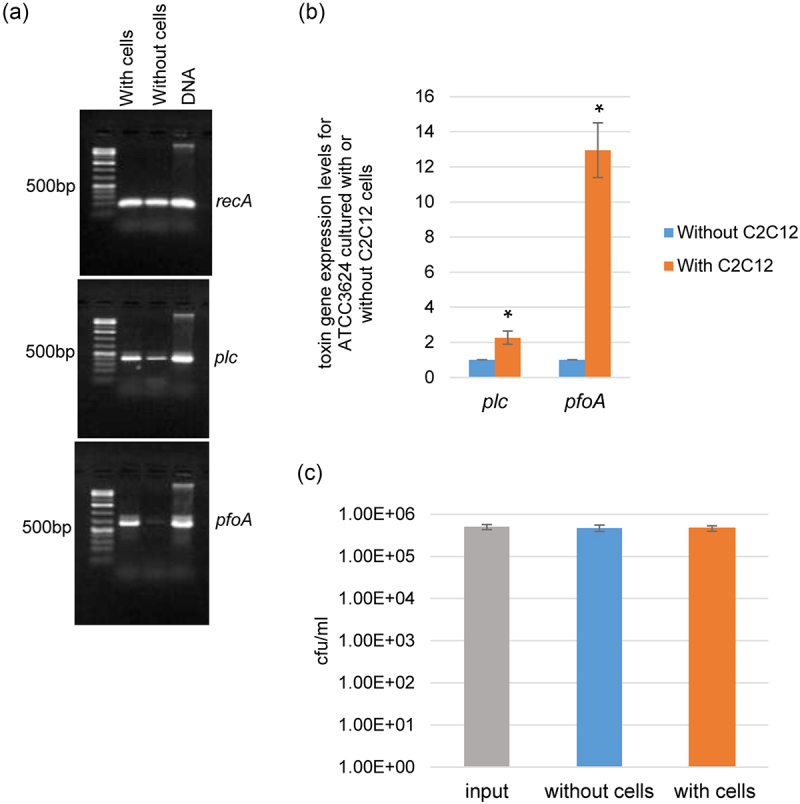
Transcription of the *plc* and *pfoA* toxin genes is upregulated when type a strain ATCC3624 is cultured in the presence of differentiated C2C12 muscle cells. Washed ATCC3624 cells were inoculated into a tissue culture dish containing HBSS with or without differentiated C2C12 cells and then incubated anaerobically for 2 h at 37 °C in a GasPak EZ pouch. Bacteria were then collected and pelleted by centrifugation. Total RNA was extracted and cDNA was made from that RNA for RT-PCR or RT-qPCR analysis. (a) RT-PCR performed with 25 ng cDNA for the housekeeping *recA* gene (top panel), the *plc* gene (middle panel) and the *pfoA* gene (bottom panel) in the presence (with cells), or absence (without cells) of differentiated C2C12 cells. Molecular markers shown in bp at left. (b) RT-qPCR for the *plc* and *pfoA* genes performed with 5 ng of the same cDNA used in panel A. Average C_T_ values were normalized to the housekeeping *recA* gene and the fold differences were calculated using the comparative C_T_ method (2^−ΔΔC^_T_). Shown are the mean values from three independent experiments. The error bars indicate the S.D. *p < .05 relative to HBSS buffer without differentiated C2C12 cells. (c) Cell culture dishes containing differentiated C2C12 cells in HBSS buffer (with cells) or HBSS only (without cells) were infected with washed ATCC3624 cells (input) and then incubated for 2 h at 37 °C. Bacteria in the culture supernatants were serially diluted and then plated onto BHI agar plates. The number of bacteria (Cfu/ml) were counted after overnight incubation under anaerobic conditions at 37 °C. Shown are the means from three independent experiments.

To model early steps in gas gangrene, our C2C12 cell infection model involved only a 2 h infection with a low dose challenge of *C. perfringens* (~10^5^ cells/ml), so Western blotting did not reliably detect PLC or PFO production. Instead, RT-PCR and RT-qPCR for toxin gene expression were used to obtain detection sensitivity with this model. Both RT-PCR (Figure 1(a)) and RT-qPCR (Figure 1(b)) analyses indicated that *plc* and *pfoA* expression levels increased when type A strain ATCC3624 was cultured in the presence of differentiated C2C12 cells. This increase in toxin gene expression levels was not due to more bacterial growth in the presence of host cells during this brief experiment since plate counting results (Figure 1(c)) showed that the number of viable input bacteria was similar to the number of viable bacteria present after this 2 h culture of ATCC3624 in the presence or absence of differentiated C2C12 cells.

### The presence of differentiated C2C12 muscle cells induces ATCC3624 to increase expression of genes encoding components of the Agr-like QSS, VirS/R TCRS, and EutV/W TCRS

**Figure 2.**
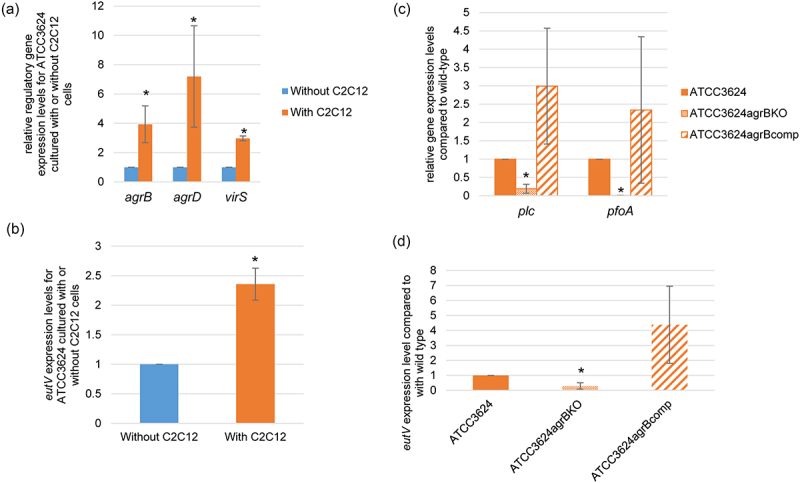
Upregulation of regulatory gene expression in the presence of differentiated C2C12 muscle cells. Washed cells of specified *C. perfringens* strains were inoculated into a cell culture dish containing HBSS or HBSS with differentiated C2C12 cells and then incubated anaerobically for 2 h at 37 °C in a GasPak EZ pouch. The bacteria were then collected and pelleted by centrifugation. Total RNA was extracted from the pellet and used to prepare cDNA for RT-qPCR analysis. Average C_T_ values were normalized to the housekeeping *recA* gene and the fold differences were calculated using the comparative C_T_ method (2^−ΔΔC^_T_). Shown are the mean values from three independent experiments. The error bars indicate the S.D. (a) RT-qPCR (5 ng of cDNA) comparing *agrB*, *agrD* and *virS* expression by ATCC 3624 cultured in the presence or absence or differentiated C2C12 cells. *p < .05 relative to HBSS in the absence of differentiated C2C12 cells. (b) RT-qPCR (5 ng of cDNA) for *eutV* gene comparison by wild type ATCC3624 in the presence of absence of differentiated C2C12 cells. *p < .05 relative to HBSS in the absence of differentiated C2C12 cells. (c) RT-qPCR for expression of *plc* and *pfoA* was performed using cDNA from the wild-type parent, *agrB* null mutant and complementing strain after their incubation in the presence of differentiated C2C12 cells. *p < .05 relative to wild type. (d) RT-qPCR (5 ng of cDNA) comparing *eutV* expression by wild-type ATCC3624, an isogenic *agrB* null mutant and an *agrB* complementing strain in the presence of differentiated C2C12 cells. *p<.05 relative to *eutV* expression by the wild-type in the presence of differentiated C2C12 cells.

Previous studies reported that, in the presence of Caco-2 enterocyte-like cells, i) the Agr-like QSS induces an upregulation of *plc* and *pfoA* expression and ii) VirS/R TCRS regulates an increase in the production of several toxins, including PLC and PFO [14,15]. Therefore, the current study performed RT-qRCR to compare *agrB/D* and *virS* expression levels when type A strain ATCC3624 is cultured in the presence vs. absence of differentiated C2C12 muscle cells. The results (Figure 2(a)) indicated that, within 2 h, the presence of differentiated C2C12 cells caused a significant increase in expression levels of those three genes encoding components of the coupled Agr-like QSS or VirS/R TCRS regulatory systems. These findings suggested possible involvement of the Agr-like QSS and VirS/R TCRS in the increase in both *plc* and *pfoA* expression that was observed in Figure 1 experiments when ATCC3624 was cultured in the presence of differentiated C2C12 cells.

It was also previously reported that the VirS/R TCRS regulates expression of *C. perfringens* genes encoding proteins involved in EA transport and utilization and that this regulation is influenced by the culture environment [32]. Therefore, the current study used the C2C12 cell culture model system to assess whether the presence of differentiated C2C12 cells affects expression levels of *eutV*, which encodes a component of the EutV/W TCRS that regulates expression of *eut* system genes involved in EA uptake and metabolism. As shown in Figure 2(b), *eutV* RT-qPCR detected a significant increase in *eutV* expression levels within 2 h of ATCC3624 encountering these host cells.

A previously prepared ATCC3624 *agrB* null mutant and complementing strain [28] were then used to test whether this differentiated C2C12 cell-induced upregulation of *eutV* expression is modulated by the Agr-like QSS system and, by extension, its coupled VirS/VirR TCRS. First, we determined that, in the presence of differentiated C2C12 cells, both *plc* and *pfoA* expression levels were significantly lower in the *agrB* null mutant compared to the wild-type and complementing strains (Figure 2(c)). Then, we found that, under the same experimental condition, *eutV* expression is also significantly lower for the *agrB* mutant, indicating that *eutV* expression is positively controlled by the Agr-like QSS system (Figure 2(d)).

### Construction and characterization of an isogenic ATCC3624 *eutV* null mutant and complementing strain

In order to test directly whether the *eut*V/W TCRS is involved in the upregulated toxin gene expression observed in Figure 1 when ATCC3624 is cultured in the presence of differentiated C2C12 cells, an isogenic *eutV* null mutant of type A strain ATCC3624 was constructed. The *Clostridium*-modified TargeTron mutagenesis system [39] was employed to construct this null mutant, which was named ATCC3624eutVKO. After curing the intron delivery plasmid, a complementing strain named ATCC3624eutVcomp was prepared using a plasmid where the entire *eutV/W* TCRS with its own promoter was cloned into the *C. perfringens*/*E. coli* shuttle plasmid pJIR750 [31,40].

Construction of the *eutV* null mutant was confirmed using primers specific for internal *eutV* open reading frame (ORF) sequences (SFigure 1A and 1B). With wild-type ATCC3624 DNA template, a PCR product of ~500 bp was amplified. However, the same primers amplified a PCR product of ~1.4 kb using template DNA from the *eutV* null mutant, a result supporting insertion of an ~900-bp intron into the *eutV* ORF. With complementing strain template DNA, the same primers amplified a product matching the size of the wild-type *eutV* product, indicative of the presence of a wild-type copy of this gene in the complementing strain.

Using the same primer pair, RT-PCR was performed to confirm that the *eutV* mutant had lost *eutV* expression and complementation had restored *eutV* expression in 4 h TY broth cultures of the wild-type parent, the isogenic *eutV* null mutant, and the complementing strain. RT-PCR for the housekeeping gene *recA* showed that the RNA quality is good (SFigure 1C, top panel). Using the same RNA preparations, *eutV* RT-PCR confirmed that the wild-type and complementing strains express the *eutV* transcript (SFigure 1C, bottom panel). However, no wild-type *eutV* transcript was detected using RNA purified from the *eutV* null mutant strain. The larger band amplified using RNA from the *eutV* mutant corresponds to disrupted RNA containing an intron insertion, as has been observed previously for some other mutants constructed using the Targetron system [41].

An intron-specific Southern blot was performed to evaluate whether only a single intron insertion was present in the *eutV* null mutant. This Southern blot analysis (SFigure 1D detected the presence of one band in the *eutV* null mutant, confirming that only a single intron insertion had occurred. As a control, no intron hybridization band was observed using DNA from the wild-type strain. Results of growth curve experiments (SFigure 1E) showed that wild-type ATCC3624, its isogenic *eutV* null mutant (ATCC3624eutVKO) and the complementing strain (ATCC362eutVcomp) grew similarly in TY medium when cultured up to 8 h at 37 °C. Extending this analysis further to include a 24-h culture timepoint, similar culture OD_600_ values were still detected for all three strains (SFigure 1F).

### Comparison of toxin or regulatory gene expression levels when wild-type ATCC3624, its *eutV* null mutant, and a complementing strain are cultured in the presence of differentiated C2C12 muscle cells

**Figure 3.**
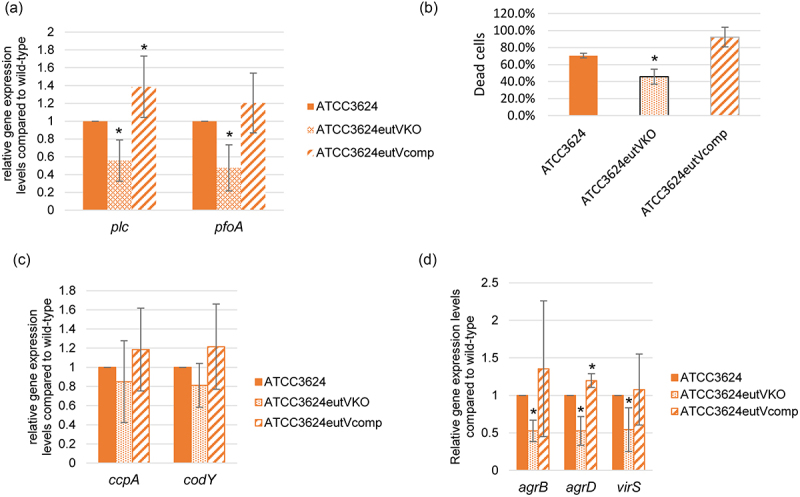
Comparison of toxin and regulatory gene expression by wild-type ATCC3624, its isogenic *eutV* null mutant and a complementing strain when incubated in the presence of differentiated C2C12 muscle cells. Washed cells of ATCC3624, the *eutV* null mutant or complementing strain were inoculated into HBSS buffer with differentiated C2C12 cells and then incubated anaerobically for 2 h at 37 °C in a GasPak EZ pouch. Bacteria were collected and pelleted by centrifugation. Total RNA was extracted from the pellet and cDNA was made for RT-qPCR analyses. Average C_T_ values were normalized to the housekeeping *recA* gene and the fold-differences in expression were calculated using the comparative C_T_ method (2^−ΔΔC^_T_). Shown are the mean values from three independent experiments. The error bars indicate the S.D. (a) RT-qPCR for expression of *plc* and *pfoA* was performed using cDNA from the wild-type parent, *eutV* null mutant and complementing strain after their incubation in the presence of differentiated C2C12 cells. *p<.05 relative to wild type. (b) Percentage of dead C2C12 cells after treatment at 37 °C for 2 h with washed cells of wild-type ATCC3624, the *eutV* null mutant and complementing strain, as assessed using the LDH cytotoxicity kit. *p<.05 relative to wild type. (c) RT-qPCRs for comparison of *ccpA* and *codY* regulatory gene expression by the wild-type parent, *eutV* null mutant and *eutV* complementing strain when incubated in the presence of differentiated C2C12 cells, *p<.05 relative to wild type with differentiated C2C12 cells. (d) RT-qPCRs to compare *agrB*, *agrD* and *virS* regulatory gene expression levels performed using RNA from wild-type ATCC3624, the *eutV* null mutant or *eutV* complementing strains incubated in the presence of differentiated C2C12 cells. *p<.05 relative to wild type with differentiated C2C12 cells.

An experiment then assessed whether the EutV/W TCRS affects toxin gene expression levels in the presence of differentiated C2C12 muscle cells. Those RT-qPCR analyses showed that, in the presence of differentiated C2C12 cells, both *plc* and *pfoA* expression levels decreased by ~50% in the *eutV* null mutant compared to the wild-type and complementing strains (Figure 3(a)). Consistent with Figure 3(a), RT-qPCR results indicating that the EutV/W TCRS positively regulates toxin gene expression in the presence of differentiated C2C12 cells, significantly less C2C12 cell cytotoxicity was detected after infection of differentiated C2C12 cell cultures with washed *eutV*KO cells compared to similar challenge with the wild-type or complementing strains (Figure 3(b)).

To explore why differences exist between relative toxin gene expression levels for wild-type ATCC3624 vs. the isogenic *eutV*KO mutant when cultured in the presence of C2C12 muscle cells, additional RT-qPCRs were performed using this C2C12 cell culture infection model. When expression of the genes encoding the CodY and CcpA global transcriptional regulators [42–45] were examined, the result showed that, in the presence of C2C12 muscle cells, no significant differences in *codY* or *ccpA* expression levels were detectable between the *eutV* null mutant vs. the wild-type or complementing strains (Figure 3(c)), i.e. changes in *codY* and *ccpA* expression levels do not explain the observed differences in toxin expression levels between the *eutV* mutant vs wild-type parent when these bacteria are cultured in the presence of differentiated C2C12 cells.

Figure 2(d) results suggested the possible involvement of the Agr-like QSS and VirS/R TCRS systems in EutV/W TCRS-mediated upregulation of toxin production in the presence of differentiated C2C12 cells. When this was examined by RT-qPCR, those analyses showed that, in the presence of differentiated C2C12 cells, expression of the *agrB*, *agrD* and *virS* genes all decreased for the *eutV* null mutant compared to the wild-type parent or complementing strain (Figure 3(d)). Since, in a coupled manner, Agr-like QSS and VirS/R TCRS positively regulate both *plc* and *pfoA* expression (Figure 2c and [21,46]), the reduced expression of genes encoding components of the Agr-like QSS and VirS/R TCRS detected in Figure 3(d) for the *eutV* mutant vs its parent strain when cultured in the presence of differentiated C2C12 muscle cells offers one explanation for the decreased toxin gene expression observed for this mutant in Figure 3(a).

### Differentiated C2C12 cell-induced upregulation of eutV gene expression can occur independently of the Agr-like QSS

**Figure 4.**
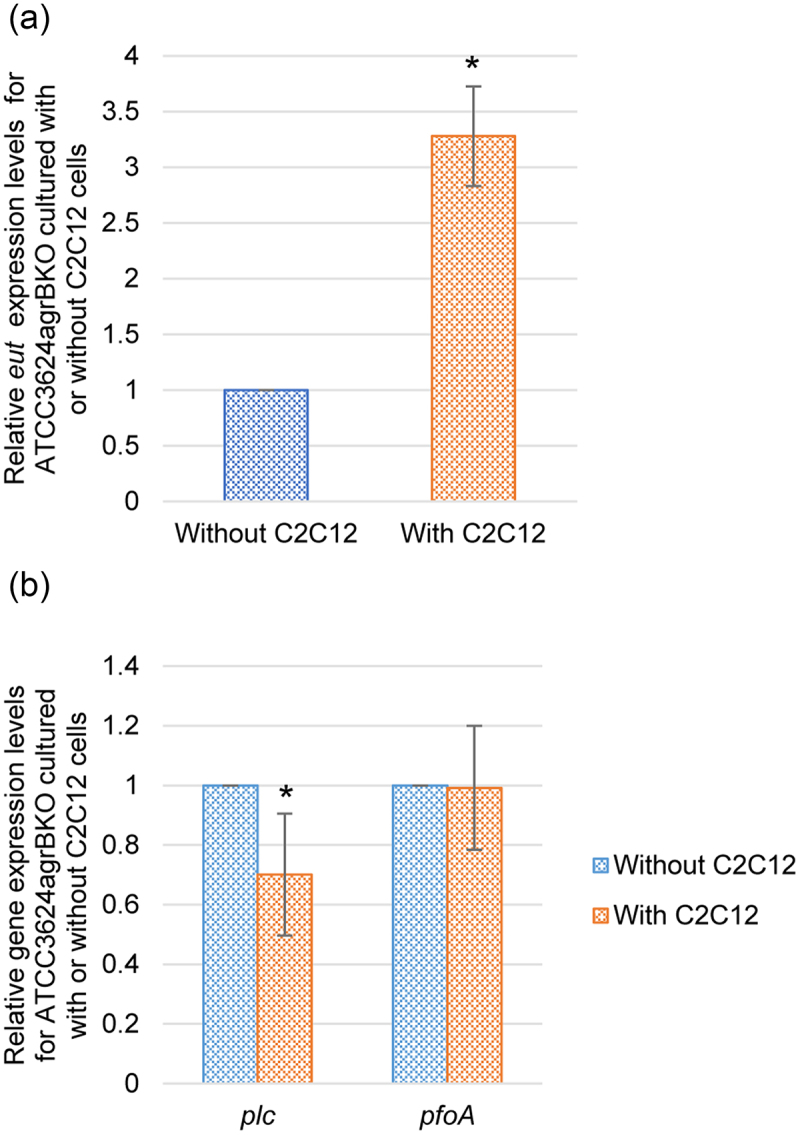
Comparison of toxin and regulatory gene expression by an ATCC3624 *agrB* null mutant cultured in the presence or absence of differentiated C2C12 muscle cells. Washed cells of the ATCC3624agrBKO mutant were inoculated into tissue culture dishes containing HBSS or HBSS with differentiated C2C12 cells and then incubated anaerobically for 2 h at 37 °C in a GasPak pouch. Bacteria were collected and pelleted by centrifugation. Total RNA was extracted from that pellet and cDNA was made for RT-qPCR analyses. Average C_T_ values were normalized to the housekeeping *recA* gene and the fold differences were calculated using the comparative C_T_ method (2^−ΔΔC^_T_). Shown are the mean values from three independent experiments. The error bars indicate the S.D. (a) RT-qPCR comparison of *eutV* gene expression by the *agrB* null mutant strain when incubated in the presence or absence of differentiated C2C12 cells. *p<.05 relative to HBSS buffer without differentiated C2C12 cells (b) RT-qPCR comparison of *plc* and *pfoA* gene expression by the *agrB* null mutant when incubated in the presence or absence of differentiated C2C12 cells. *p<.05 relative to relative to HBSS buffer without differentiated C2C12 cells.

While *eutV*, *plc* and *pfoA* expression was much lower for ATCC3624agrBKO *vs*. the wild-type parent or complementing strain in the presence of differentiated C2C12 muscle cells (Figure 2), the *agrB* null mutant still exhibited some expression of those genes in the presence of these muscle cells. Therefore, to examine whether this residual *eutV* expression by ATCC3624agrBKO mutant is C2C12 cell-induced, RT-qPCR was performed to compare *eutV* expression when this mutant was cultured in the presence vs absence of differentiated C2C12 cells. The results showed (Figure 4(a)) that differentiated C2C12 cells did induce some *eutV* expression by the *agrB* mutant, indicating that this induction does not absolutely require a functional Agr-like QSS.

Using the same cDNA preparations, RT-qPCRs for *plc* and *pfoA* expression were also performed. Unlike the *eutV* results, culture of the ATCC3624agrBKO mutant in the presence vs absence of C2C12 cell showed this mutant lost the ability to upregulate *pfoA* expression in the presence of differentiated C2C12 muscle cells (Figure 4(b)), and expression levels for *plc* expression actually decreased in these conditions (Figure 4(b)).

Collectively, Figures 2 and 4 results indicate that the upregulated *eutV* expression induced by the presence of differentiated C2C12 cells is mainly, but not completely, regulated by the Agr-like QSS system and, by extension, the VirS/R TCRS. However, the C2C12 cell-induced upregulation of *pfoA* and *plc* expression appears to require the Agr-like QSS system and, by extension, VirS/R TCRS. In addition, other regulators are involved in controlling *plc* expression in the presence of these muscle cells.

### Evidence that the Agr-like QSS and VirS/R TCRS can function independently of the EutV/W TCRS

Since Figure 4 results indicated that, in the presence of differentiated C2C12 cells, some upregulation of *eutV* expression still occurs independently of the Agr-like QSS, the reverse experiment was performed, i.e. in the presence of differentiated C2C12 cells, is some expression of genes encoding Agr-like QSS and VirS/R TCRS components upregulated independently of EutV/W TCRS?

**Figure 5.**
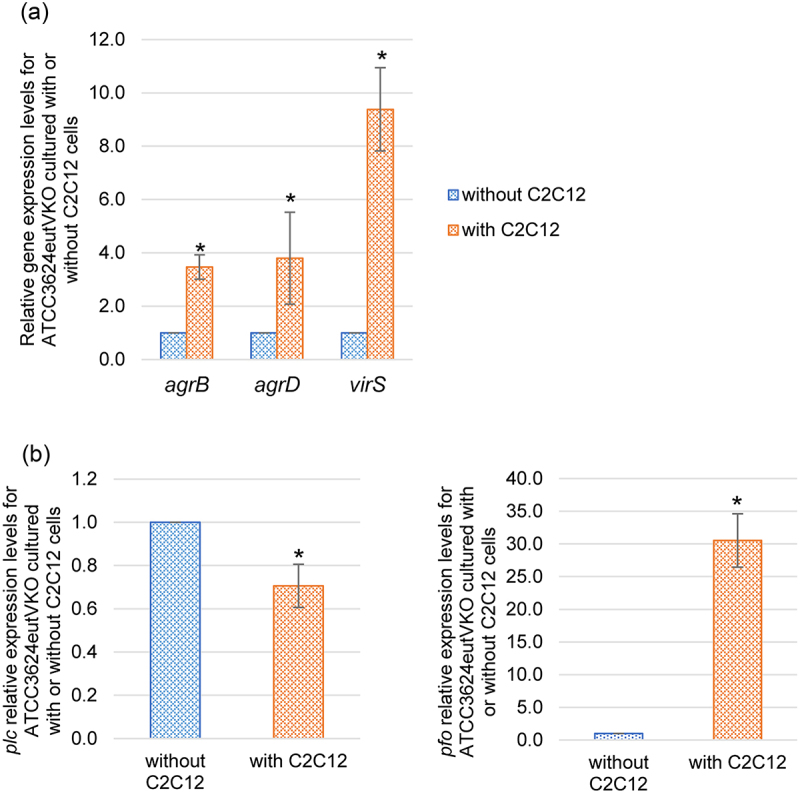
Comparison of toxin and regulatory gene expression by an ATCC3624 *eutV* null mutant cultured in the presence or absence of differentiated C2C12 muscle cells. Washed cells of the ATCC3624eutVKO mutant were inoculated into cell culture dishes containing HBSS or HBSS with differentiated C2C12 cells and then incubated anaerobically for 2 h at 37 °C in a GasPak pouch. Bacteria were collected and pelleted by centrifugation. Total RNA was extracted from the pellet and cDNA was made for RT-qPCR analyses. Average C_T_ values were normalized to the housekeeping *recA* gene and the fold differences were calculated using the comparative C_T_ method (2^−ΔΔC^_T_). Shown are the mean values from three independent experiments. The error bars indicate the S.D. (a) RT-qPCR comparison of regulatory gene (*agrB*, *agrD* or *virS*) expression by the *eutV* null mutant cultured in the presence or absence of differentiated C2C12 cells. *p<.05 relative to HBSS without differentiated C2C12 cells (b) RT-qPCR comparison of *plc* (left panel) and *pfoA* (right panel) expression by the *eutV* null mutant cultured in the presence or absence of differentiated C2C12 cells *p<.05 relative to relative to HBSS buffer without differentiated C2C12 cells.

For this purpose, RT-qPCRs were performed using cDNA from ATCC3624*eutV*KO cultured in the presence or absence of differentiated C2C12 cells, and *agrB*, *agrD* and *virS* expression levels were compared under these two culture conditions. The results indicated that upregulated expression of genes encoding Agr-like QSS and VirS/R TCRS in the presence of differentiated C2C12 cells is not strictly dependent on the EutV/W TCRS (Figure 5(a)).

The same cDNAs were also used to assess whether the ATCC3624*eutV*KO mutant still shows some upregulation of *plc* and *pfoA* expression in the presence vs absence of C2C12 cells. Relative to culture in the absence of differentiated C2C12 cells, *pfoA* expression by ATCC362*eutV*KO still increased in the presence of differentiated C2C12 cells, indicating that some upregulation of *pfoA* expression can still occur in the absence of *eutV*. However, resembling the response of the ATCC3624agrBKO mutant to differentiated C2C12 cells, there was reduced *plc* expression when ATCC3624*eutV*KO was cultured in the presence vs absence of differentiated C2C12 muscle cells (Figure 5(b)).

### Extracellular EA induces *eutV*, *pfoA* and *plc* expression

**Figure 6.**
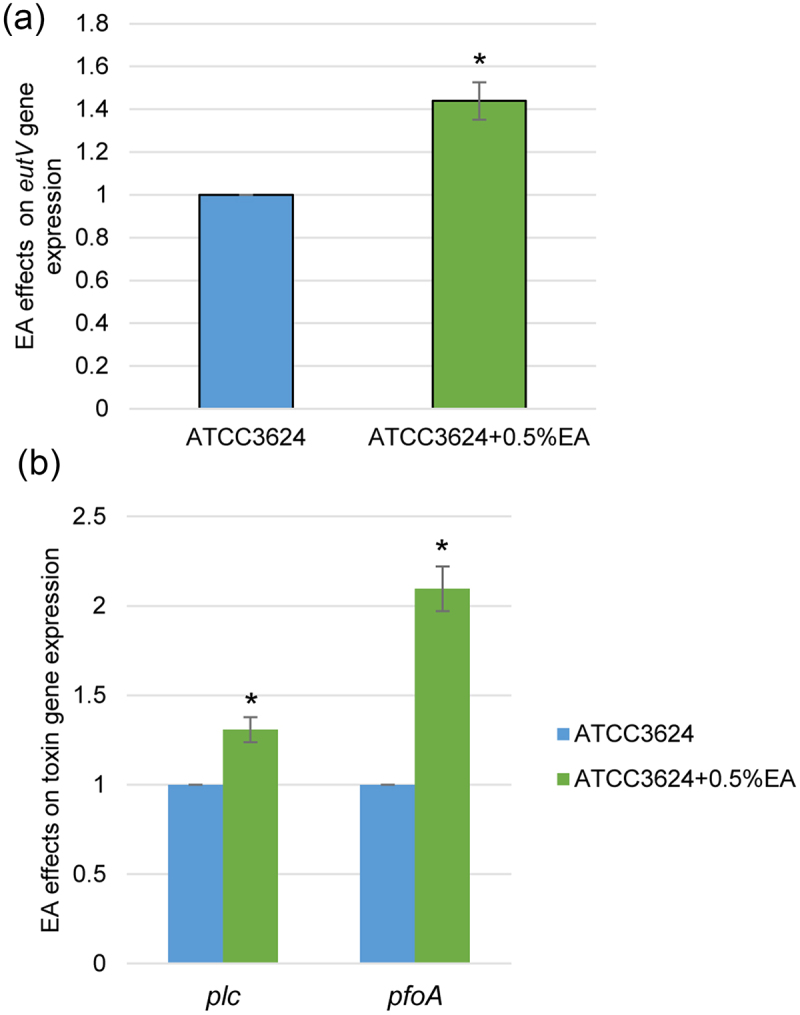
Effects of EA on toxin and regulatory gene expression by wild-type ATCC3624. Washed cells of ATCC3624 were inoculated into cell culture dishes containing HBSS with or without 0.5% EA and those cultures were then incubated anaerobically for 1 h at 37 °C in a GasPak pouch. Bacteria were collected and pelleted by centrifugation. Total RNA was extracted from the pellet and cDNA was made for RT-qPCR analysis. Average C_T_ values were normalized to the housekeeping *recA* gene and the fold differences were calculated using the comparative C_T_ method (2^−ΔΔC^_T_). Shown are the mean values from three independent experiments. The error bars indicate the S.D. (a) RT-qPCR comparison of *eutV* expression in the presence or absence of EA. *p<.05 relative to HBSS without 0.5% EA. (b) RT-qPCRs comparison of *plc* and *pfoA* expression in the presence or absence of EA. *p<.05 relative to HBSS without 0.5% EA.

This study then evaluated whether EA affects *eutV* expression in ATCC3624 by performing *eutV* RT-qPCR after the addition of 0.5% EA to HBSS buffer. Compared to culture with HBSS buffer itself (no EA), the presence of 0.5% EA significantly increased *eutV* gene expression for wild-type ATCC3624 after a 1 h incubation at 37 °C (Figure 6(a)).

To assess whether EA also affects toxin gene expression levels by ATCC3624, *plc* and *pfoA* RT-qPCR were performed with the same cDNA used for Figure 6(a). The results showed that expression of both toxin genes significantly increased in the presence of EA (Figure 6(b)). Therefore, by inducing *eutV/W* TCRS expression, EA is one host cell nutrient that can increase toxin genes expression when this type A strain is cultured in the presence of muscle cells.

### Construction and characterization of a *plc* null mutant, a *pfoA* null mutant, and a *plc/pfoA* double null mutant of ATCC3624, as well as complementing strains for the single *plc* and *pfoA* null mutants

This study next wanted to explore what is the benefit to ATCC3624 of increasing toxin production in the presence of C2C12 muscle cells, as demonstrated in this study? To address this question, an isogenic *plc* null mutant (ATCC3624*plc*KO) and an isogenic *pfoA* null mutant (ATCC3624*pfoA*KO) of type A strain ATCC3624 were constructed. In addition, the *pfoA* gene in ATCC3624plcKO was inactivated to create a *plc/pfoA* double null mutant strain (ATCC3624DKO).

The Targetron mutagenesis system [39] was used to construct these toxin mutants. Using primers specific for internal *plc* or *pfoA* ORF sequences, wild-type ATCC3624 DNA supported PCR amplification of an ~400 bp product for *plc* and an ~650 bp product for *pfoA* (SFigure 2A to C). However, the same primers PCR-amplified an ~1.3 kb product using DNA from the *plc* null mutant or an ~1.5 kb product using DNA from the putative *pfoA* null mutant. Those results are consistent with the insertion of an ~0.9 kb intron into the *plc* or *pfoA* genes, respectively, in the two mutants. DNA isolated from the *plc/pfoA* double null mutant strain supported PCR amplification of both ~1.3 kb and ~1.5 kb products, indicative of intron insertion into both the *plc* and *pfoA* genes.

After curing the intron delivery plasmids in the single *plc* or *pfoA* null mutants, two complementing strains, named ATCC3624*plc*comp and ATCC3624*pfoA*comp, were prepared by transformation of the relevant single toxin mutant with plasmids containing the *plc* ORF or *pfoA* ORF, and their upstream sequences were cloned into the *C. perfringens*/*E. coli* shuttle plasmid pJIR750. DNA from the *plc* complementing strain supported amplification of a product matching the ~0.4 kb size of the *plc* product amplified from wild-type ATCC3624, while DNA from the *pfoA* complementing strain supported amplification of a product matching the ~0.65 kb size of the *pfoA* product amplified from wild-type ATCC3624. Note that it is not technologically possible at present to complement a double null mutant in *C. perfringens.*

Southern blot analysis (SFigure 2D) indicated the presence of only a single intron insertion in either the single *plc* or *pfoA* null mutants. However, as expected, two intron insertions were detected in the double null mutant strain. As a control, no hybridization of the intron was detected using DNA from the wild-type strain. When the toxin null mutants and complementing strains became available, the growth of these strains was compared in TY medium. When cultured at 37 °C in TY medium, all six strains grew very similarly (SFigure 2E), even after 24 h of culture (SFigure 2F).

Using supernatants from those 24 h TY cultures (where there is much stronger toxin production than with our 2 h C2C12 cell infection model), PLC and PFO Western blotting (SFigure 2 G) confirmed that the single toxin null mutant strains did not produce toxin from their inactivated toxin gene. Furthermore, these Western blots demonstrated that the complementing strains recovered toxin production. Lastly, the double null mutant did not produce any PLC or PFO, as expected.

### ATCC3624 grows better than ATCC3624DKO in the presence of differentiated C2C12 cells

A previously described Transwell 0.4-µm-pore-size membrane filter support model was used to explore whether toxin production in the presence of differentiated C2C12 cells might affect the growth of ATCC3624 [47]. In this model, the lower chamber of same Transwells contained HBSS buffer covering a monolayer of differentiated C2C12 muscle cells. As a control, the lower chamber of other wells contained only HBSS buffer. The upper chamber of all Transwells contained HBSS with 2.5% Oxyrase.

**Figure 7.**
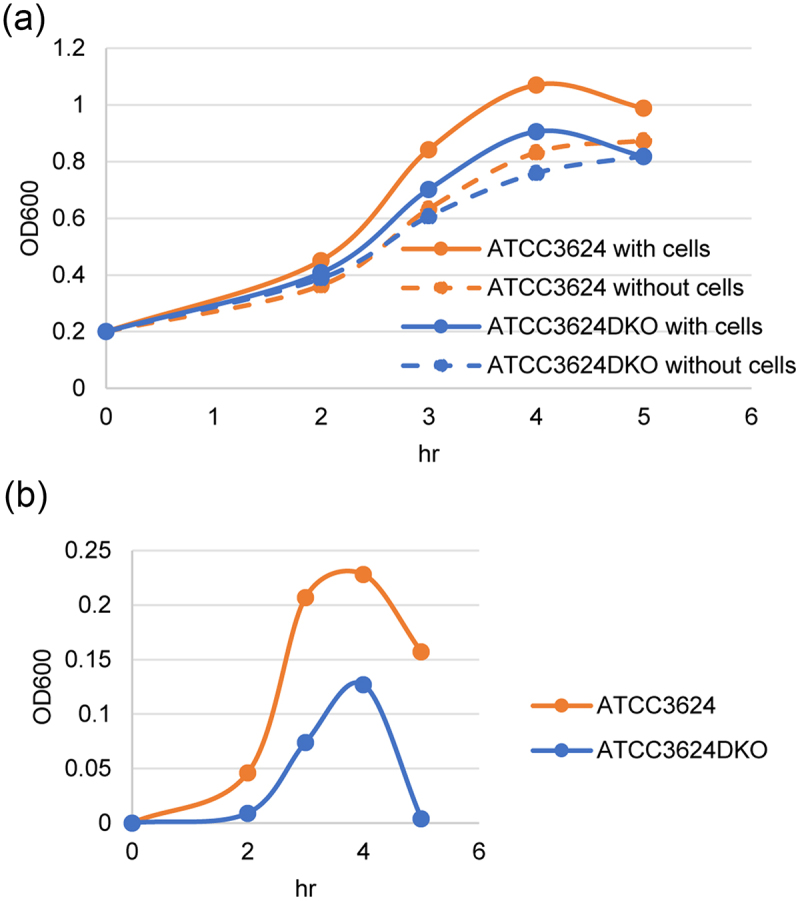
Comparison of growth when ATCC3624 and its *plc/pfoA* double null mutant are cultured in HBSS buffer with or without differentiated C2C12 cells. (a) Post-inoculation changes in the OD_600_ for cultures of wild-type ATCC3624 or its isogenic double null mutant when cultured anaerobically at 37° in cell culture dishes containing HBSS or HBSS with differentiated C2C12 cells. At 2 h intervals, up to 5 h, a 0.6-ml aliquot of the upper chamber was removed for OD_600_ determination. (b) Adjusted growth curve for the wild-type parent and double toxin null mutant. The post-inoculation changes in OD_600_ shown reflects the OD_600_ measured in the presence of differentiated C2C12 cells minus the OD_600_ measured in the absence of differentiated C2C12 cells. The results shown are representative of three repetitions.

To start evaluating the effects of toxin production on ATCC3624 growth in the presence of C2C12 cells, the wild-type parent or an isogenic *plc*/*pfoA* double null mutant were added to the upper chamber of these Transwells for 2, 3, 4, or 5 h. The resultant OD_600_ growth curve results are shown in Figure 7(a). Those results indicated that wild-type ATCC3624 and the double toxin null mutant both grew better in the presence of differentiated C2C12 cells than in their absence. However, the wild-type parent grew more than the double toxin null mutant in the presence of differentiated C2C12 cells, suggesting that toxin production may impact ATCC3624 growth. Both ATCC3624 and its double toxin mutant grew less, although similarly, in the absence of differentiated C2C12 cells. An adjusted growth curve is shown in Figure 7(b), which depicts the OD_600_ in the presence of differentiated C2C12 cells minus the OD_600_ without cells. This adjusted growth curve shows the specific impact of differentiated C2C12 cells on growth of both strains and indicates that this effect peaks at 4 h of culture. These results suggested a role for toxins during the enhanced growth of ATCC3624 in the presence of differentiated C2C12 cells.

### Comparison of PFO vs. PLC contributions to growth and cytotoxicity of ATCC3624 when cultured in the presence of differentiated C2C12 cells

A final experiment was performed using the single toxin null mutants to determine definitively the contributions of PFO vs. PLC to 3 or 4 h (peak) growth and the 4 h cytotoxic activity of ATCC3624 in the presence of differentiated C2C12 cells.

**Figure 8.**
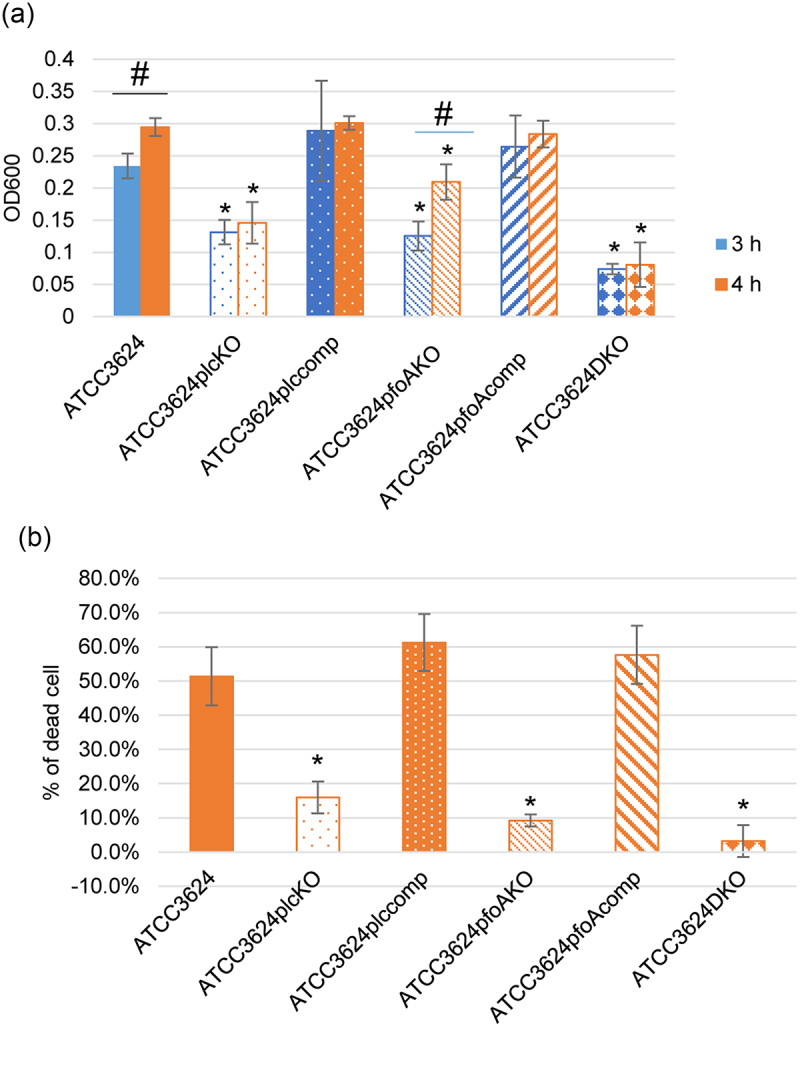
Comparison of growth and cytotoxicity of wild-type ATCC3624 and its isogenic *plc*, *pfoA*, *plc/pfoA* null mutants, as well as *plc* or *pfoA* complementing strains (a) adjusted post-inoculation changes in OD_600_ after anaerobic culture at 37 °C for 3 or 4 h for the wild-type parent, its toxin null mutants and complementing strains. Adjusted values were calculated as OD_600_ when cultured in HBSS with differentiated C2C12 cells minus the OD_600_ for those strains cultured in HBSS without differentiated C2C12 cells. Shown are the mean values from three independent experiments. The error bars indicate the S.D. *p<.05 relative to wild type. # *p* < .05 relative to 3 h (b) percentage of dead C2C12 cells in panel a cultures after treatment at 37 °C for 4 h (samples from panel a bottom chambers) using the LDH cytotoxicity kit. The error bars indicate the S.D. *p<.05 relative to wild type.

The adjusted OD_600_ (i.e. the culture OD_600_ after subtraction of OD_600_ values for the strain cultured in the absence of differentiated C2C12 cells from the OD_600_ values for the same strain cultured in the presence of differentiated C2C12 cells) showed that cultures of the ATCC3624*plc*KO, ATCC3624*pfoA*KO and ATCC3624DKO mutants all grew less than wild-type ATCC3624 or the complementing strains for the single toxin mutants at either time-point (Figure 8(a)). Only the wild-type parent and *pfoA*KO strains showed a significant OD_600_ increase between 3 and 4 h of culture due to the presence of differentiated C2C12 cells. These results indicated that both PLC and PFO, but particularly PLC, are useful for obtaining host cell nutrients to support *C. perfringens* growth.

Supporting this conclusion, 4 hr cytotoxicity results showed that ATCC3624plcKO, ATCC3624*pfoA*KO and ATCC3624DKO caused less C2C12 cell cytotoxicity than wild-type ATCC3624 or the single complementing strains (Figure 8(b)).

## Discussion

*Clostridium perfringens* ranks among the most prolific bacterial producers of toxins, with >20 different *C. perfringens* toxins reported in the literature [1,3]. It has been experimentally established that two of those toxins, i.e. PLC and PFO, are important when this bacterium causes histotoxic infections, such as gas gangrene, or infections originating in the intestines, such as *C. perfringens* type F food poisoning [11,48–50].

When causing disease, *C. perfringens* benefits in several proven ways from its abundant toxin production. For example, by producing toxins (such as *C. perfringens* enterotoxin or CPB) active in the gastrointestinal tract, type F or C strains (respectively) of this bacterium induce diarrhoea, which then likely promotes their dissemination from the host. During gas gangrene caused by type A strains, PLC and PFO reduce vascular flow, which causes tissue hypoxia that facilitates *C. perfringens* growth [9]. Those two toxins also inhibit the recruitment of immune cells to the site of infection, thereby contributing to the establishment and spread of gas gangrene [1,9].

Another potential, but little studied, benefit for producing these toxins has also been long postulated, i.e. some of these toxins may release substrates from host cells, which *C. perfringens* can then utilize for growth during disease. If this proposal is correct, using toxins to obtain nutrients from host cells should be particularly important for *C. perfringens* growth during gas gangrene, where impaired vascular flow would make it difficult or impossible to obtain sufficient substrates from blood to support continued *C. perfringens* growth [9].

Therefore, an important contribution of this study is providing the first evidence (to our knowledge) that toxins can promote growth of *C. perfringens* in the presence of host cells, including when a type A strain encounters a differentiated C2C12 muscle cell line, which is a relevant *in vitro* model for studying gas gangrene pathogenesis. While other yet to be identified factors may also be involved, this study established that both PLC and PFO significantly contribute to *C. perfringens* growth in the presence of differentiated C2C12 muscle cells. These toxin contributions to growth likely reflect the ability of both PLC and PFO to damage the host cell membrane. PLC is a phospholipase C that hydrolyzes membrane phospholipid, while PFO forms very large pores in host cells that allow release of cytoplasmic contents [3,8,9]. Both such effects should release critical nutrients from muscle cells to support *C. perfringens* growth during gas gangrene.

Consistent with the finding that *C. perfringens* can grow by utilizing toxin-induced release of growth substrates from differentiated C2C12 cells, the current study also demonstrated that the cytotoxic activity of type A strain ATCC3624 increases in the presence of these differentiated muscle cells. This effect involved a C2C12 cell-induced upregulation of *plc* and *pfoA* expression. In agreement with previous reports that the Agr-like QSS and VirS/R TCRS regulate *plc* and *pfoA* expression in laboratory media [15,17–25], C2C12 cell-induced upregulation of *plc* and *pfoA* expression was also shown to involve this coupled regulatory system.

Furthermore, this study determined that the C2C12 cell-induced increase in *plc* and *pfoA* expression also involves regulation by the EutV/W TCRS, although this regulation was less strong than for Agr-like QSS and VirS/R TCRS. EutV/W controls expression of the 19 gene *eut* system that mediates EA uptake and utilization of ethanolamine by *C. perfringens* [32]. This is pathogenicity-relevant because mammalian cell membranes contain abundant phosphatidylethanolamine, which can be broken down by PLC to glycerol and EA [32]. EA can then be transported into *C. perfringens*, where it can be used for its metabolic and energy needs [31,32]. The current study showed that the addition of EA to HBSS significantly increased *plc* and *pfoA* expression by ATCC3624, suggesting that EA is one trigger (there may be others) used by type A strains to sense the presence of muscle cells, i.e. during gas gangrene, sensing of EA signals this bacterium to its presence in an environment containing muscle cells, so it becomes beneficial to increase toxin production for obtaining more nutrients from those host cells. EutV/W TCRS upregulation of toxin production in the presence of muscle cells may help to explain previous results indicating that an *eutV* null mutant is attenuated for causing gas gangrene in a mouse model [31]. Lastly, the current study also identified a mechanism behind EA triggering of increased toxin gene expression, i.e. this effect likely involves an EA-induced increase in *eutV/W* transcription.

Interestingly, in the presence of differentiated C2C12 cells, expression of both *agrB/D* and *virS* significantly decreased for the *eutV* null mutant compared to it wild-type parent or complementing strain. Since the Agr-like QSS and coupled VirS/R TCRS are involved in controlling expression of *plc* and *pfoA* in the presence of C2C12 cells, this reduced *agrB/D* and *virS* expression by the *eutV* null mutant likely helps to explain why, relative to ATCC3624 or the complementing strain, expression levels of *plc* and *pfoA* decreased for the *eutV* mutant, so this mutant caused less C2C12 cell cytotoxicity.

Another finding of this work is that both the *eutV/W* TCRS and *agrB/D QSS-virS/R* TCRS cross-regulate expression of one another in the presence of differentiated C2C12 cells. Neither the *agrB* null mutant nor *eutV* null mutant completely lost responsiveness to the presence of differentiated C2C12 cells with respect to upregulating, respectively, their *eutV* or *agrB* expression, although their toxin gene expression response to differentiated C2C12 cells did show some alterations. For the *agrB* null mutant, *pfoA* expression levels did not increase in the presence of differentiated C2C12 cells, although there was still some C2C12 cell-induced increase in *pfoA* expression for the *eutV* mutant under this condition. Expression of *plc* decreased for both the *agrB* and *eutV* null mutants cultured in the presence of differentiated C2C12 cells. These results confirmed previous reports that VirS/R TCRS regulates *plc* and *pfoA* expression [18,19,26] and also demonstrated that the EutV/W TCRS directly or indirectly upregulates *plc* expression in the presence of differentiated C2C12 cells.

**Figure 9.**
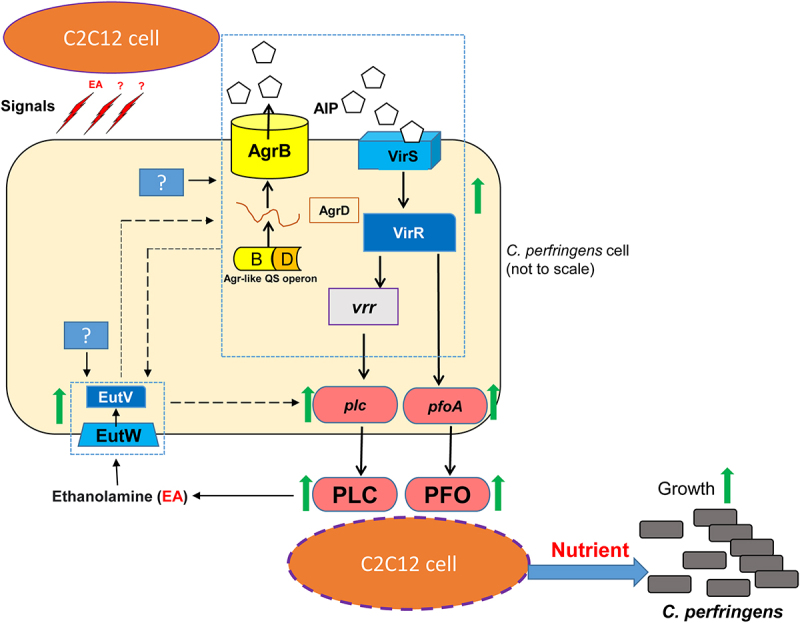
Model for regulation of expression of the *C. perfringens* type A toxin genes. When *C. perfringens* type A encounters muscle cells, its basal level of toxin production produces signal(s) (likely including EA and possibly other host substances) that causes this bacterium to rapidly upregulate toxin (PLC and PFO) production. This involves increased toxin gene mediated by the Agr-like QSS coupled with the VirS/R TCRS. Phosphorylated VirR directly increases *pfoA* gene expression and indirectly increases *plc* gene expression. In addition, EA increases expression of the *eutV/W* TCRS genes, which also results in directly or indirectly upregulated *plc* and *pfoA* expression. The EutV/W TCRS increases expression of genes encoding the Agr-like QSS and VirS/R TCRS and vice versa. Increased toxin production generates more EA, which further increases *eutV/W* TCRS expression that, via a feedback loop, leads to even more toxin gene expression. The presence of increasing levels of EA and EutV/W TCRS also leads to more expression of the *eut* system genes, which allows increased uptake and utilization of EA for increased growth. That effect and, likely, increased release of other nutrients from differentiated C2C12 cells provides *C. perfringens* with more nutrients to support growth. “?” unknown regulatory factors. Solid lines show direct regulation and dashed lines show regulation that may be either direct or indirect, but this is not yet distinguished.

Combining the current and previous results suggests a model (Figure 9) to explain events occurring when a type A strain encounters C2C12 muscle cells. The type A strain initially produces low levels of toxins, including PLC, which generate some EA. This EA provides one signal (there may be others) that the type A strain is in the presence of muscle cells, making it beneficial to increase expression of the *eutV/W* TCRS genes, the *agr-*like QSS genes, and the *virS/R* TCRS genes. This upregulation results in the coupled Agr-like QSS and VirS/R TCRS directly increasing PFO production and indirectly increasing PLC production; the EutV/W TCRS also directly or indirectly increases toxin production. In addition, the EutV/W TCRS and the coupled Agr-like QSS and VirS/R TCRS cross-upregulate expression levels of one another. This increased production of toxins results in release of even greater amounts of EA (and perhaps other mammalian cell signals) from muscle cells. This effect further increases *eutV/W* expression, which continues the feedback loop by enhancing toxin production even more. In addition to liberating EA, this increased toxin production likely damages host cell plasma membrane permeability to release increasing amounts of additional nutrients from differentiated C2C12 cells. Collectively, these released nutrients boost growth of type A strains.

The demonstrated involvement of EutV/W and VirS/R in sensing the presence of muscle cells and regulating production of PFO or PLC in the presence of muscle cells does not necessarily exclude the possible involvement of other *C. perfringens* TCRs in sensing host cells or regulating toxin gene expression. It is also possible that, besides PLC and PFO, other secreted *C. perfringens* substances, such as exoenzymes, may further contribute to these sensing and induction events. The role of other *C. perfringens* TCRS and secreted substances in sensing and liberating nutrients, from muscle cells should be studied in the future.

## Materials and methods

### Bacteria, media, and chemicals

*C. perfringens* type A strain ATCC3624 was purchased from ATCC. *Escherichia coli* DH5α competent cells were purchased from New England Biolabs (NEB). *C. perfringens* was cultured using fluid thioglycolate medium (FTG) (Difco Laboratories); TY broth (3% tryptic soy broth [Becton-Dickinson], 1% yeast extract [Becton Dickinson], and 0.1% sodium thioglycolate [Sigma-Aldrich]) or TGY broth (TY broth supplemented with 2% glucose [Sigma-Aldrich]). For constructing isogenic null mutants and complementing strains, Brain Heart Infusion (BHI) agar (Research Products International) plates containing 15 μg ml^−1^ chloramphenicol (CM, Sigma-Aldrich) was used. To culture *E. coli*, Luria-Bertani, Miller (LB) broth or LB agar (Fisher Scientific) were used. For anaerobic culture of *C. perfringens*, GasPak jars and packets were purchased from Fisher Scientific.

All other chemical reagents used in this study were purchased from Fisher Scientific, Sigma Aldrich, or Bio-Rad.

### Primers and plasmids

Primers were designed by Vector NTI or Integrated DNA Technologies (IDT). All primers were synthesized by IDT and are listed in Table 1. The plasmid vector used in this study was the *E. coli-C. perfringens* shuttle plasmid pJIR750 [40]. The *eutV/W* null mutant of ATCC3624 was constructed using pJIR750eutVi and *Clostridium*-modified group II TargeTron^@^ Technology [39]. After transformation of ATCC3624 by electroporation, the intron on this newly-constructed plasmid inserted (in the sense orientation) into the *eutV* ORF of *eutV* between nucleotides 297 and 298. The primers used to construct pJIR750eutVi are listed in Table 1. The 350-bp intron PCR product amplified with those primers was inserted into pJIR750ai [39] between the HindIII and BsrGI enzyme sites. The single and double *plc* and *pfoA* null mutant strains were prepared using pJIR750ai and pJIR750pfoAi vectors [39,48]. All null mutant screening primers used to verify these null mutants are listed in Table 1. The complementation vectors pJIR750eutVcomp, pJIR750plccomp and pJIRpfoAcomp were also constructed using the pJIR750 shuttle plasmid. The enzyme and primer sequences used for this purpose are listed in Table 1. All RT-qRCR primers for assessing expression of the *plc*, *pfoA*, *agrB*, *agrD*, *virS*, *and eutV* genes are also listed in Table 1. The *recA* gene served as a control housekeeping gene for RT-qPCR experiments and the *recA* primer sequences [51] used are listed in Table 1.

**Table 1.** Primers used in this study.

primer	sequence	size	purpose
eutV-297s-IBS	AAAAAAGCTTATAATTATCCTTAGTAGGCGCCTTT GTGCGCCCAGATAGGGTG	350 bp	*eutV* null mutant plasmid (pJIR750eutVi)
eutV-297s-EBS1d	CAGATTGTACAAATGTGGTGATAACAGATAAGTCG CCTTTGGTAACTTACCTTTCTTTGT		
eutV-297s-EBS2	TGAACGCAAGTTTCTAATTTCGATTCCTACTCGAT AGAGGAAAGTGTCT		
EBS universal	CGAAATTAGAAACTTGCGTTCAGTAAAC		
eutVKOF	ATAGTCGATGATGAGCCTATTACAA	439 bp (WT) 1339 bp (KO)	*eutV* null mutant screening
eutVKOR	CAAGTATTCCTTTTGCCTTCTCTA		
eutVcompF	CCGGggatccAAGCTTCAAAATAGGACTGATATGC (BamHI)	3601 bp	*eutV/W* complementing plasmid (pJIR750eutVcomp)
eutVcompR	ATGCctgcagCAGCCTTTTTATATTCTTCTTCAAT (PstI)		
pfoAKOF	TTTATGAACTTAACAAATGAGGGG	678 bp (WT) 1578 bp (KO)	*pfoA* null mutant screening, RT-PCR
pfoAKOR	CTACTCCAAGTGAGTTTTCAAGG		
pfoAcomF	TCATgaattcAGTTGTAAGTGCAGGTAGTGGATAT (EcoRI)	2409 bp	*pfoA* complementing plasmid (pJIR750pfoAcomp)
pfoAcomR	TCATgaattcAATGCCTCAGCTAATTTCATTTT (EcoRI)		
plcKOF	GATTTGTAAGGCGCTTATTTGTG	400 bp (WT) 1300 bp (KO)	*plc* null mutant screening, RT-PCR
plcKOR	CCATTCATATCTAGCTAATGCTG		
plccompF	TGTAGGAATTCCAAGACCATGCATACC (EcoRI)	2047 bp	*plc* complementing plasmid (pJIR750plccomp)
plccomR	TTCCTAAGCTTTTATTTTGTAAATACC (HindIII)		
recAF	CTGGTAAAACAACAGTGGCTTT	167 bp	RT-PCR and RT-qPCR for *recA* house-keeping gene [51]
recAR	AGCTTGTTCTCCTGTATCTGGT		
plcqF	ATAGCGCAGGACATGTTAAGT	90 bp	*plc* RT-qPCR
plcqR	CTTAACATGTCCTGCGCTATCA		
pfoAqF	CAGTTGCTGCTGTTCACAATAA	131 bp	*pfoA* RT-qPCR
pfoSqR	TGAAACTTCATCCCATGCTACT		
eutWqF	AAGAGAAGCTCATCATAGGGTTAAA	108 bp	*eutV* RT-qRCR
eutWqR	TCCTGTAAATATTCCTTAGCCTCTTC		
ccpAqF	ACACATAGAACAAGAACTGTAGGT	87 bp	*ccpA* RT-qPCR
ccpAqR	TACGTCCTCAGCACCTCTAA		
codYqF	GTGCTACAATAGTTGGGATGGA	94 bp	*codY* RT-qPCR
codYqR	CCAATAGCTAATTGAACCACTGC		
agrBqF	GGAGCACATTCAGAATCCTCTAA	126 bp	*agrB* RT-qPCR
agrBqR	TCCAATAAACACTACAAGCTCAAAG		
agrDqF	GCTGCATTAACAACAGTAGTTGC	76 bp	*agrD* RT-qPCR
agrDqR	GTTCCTCTGGTTGGTGTGTAAA		
virSqF	CATAGCCTGTATTGAAGGAAATAAC	229 bp	*virS* RT-qPCR
virSqR	TGTGCAGATATCAAAGTACTCA		

### Mouse muscle C2C12 cell culture

The authenticated C2C12 cell line was purchased from Sigma Aldrich. Undifferentiated C2C12 muscle cells were grown utilizing 500 ml of DMEM high glucose (Sigma D5796), supplemented with 75 ml of Fetal Bovine Serum (FBS,Gibco) and 5 ml of 100× Penicillin-Streptomycin solution (Corning). Differentiation of C2C12 cells was induced using 500 ml of DMEM high glucose, with 10 ml of horse serum (Fisher Scientific) and 5 ml of 100× Penicillin–Streptomycin solution.

To prepare C2C12 cell cultures, a frozen vial of C2C12 myoblasts (Sigma) was thawed and transferred to a plate with C2C12 growth medium. Those cell culture plates were grown at 37 °C in 5% CO2 and then subcultured every 2–3 d until the cultures reached 70% confluency. Differentiation medium was then added, and the cultures were further incubated under the same conditions. Every 2 d, the differentiation medium was changed until day 6 when the cultures were used for experiments.

### *C.* *perfringens* DNA isolation, PCR, and Southern blot analyses

DNA was obtained using the MasterPure Gram-Positive DNA purification kit (Epicenter), according to the manufacturer’s instructions. For each strain, 3.0 ml of an overnight TGY culture pellet was used. Purified DNA was suspended in 50 µl of ddH_2_O. PCR primers used are listed in Table 1. PCR used DreamTaq Green PCR Master Mix (2X) (Fisher Scientific) and LongAmp® Taq 2X Mix (New England Biolabs). Annealing temperature was 55 °C, with an extension time of 1 min per kb, for a total of 35 cycles.

For Southern blot analyses, a 4 µg aliquot of each DNA was first digested overnight with EcoRI at 37 °C (New England Biolabs). Each digested DNA was electrophoresed on a 1% agarose gel before transfer to a positively charged nylon membrane (Roche). Those blots were then hybridized with an intron-specific probe, which had been prepared with the PCR DIG Probe Synthesis Kit (Roche). All protocols were prepared according to the manufacturer’s instructions.

### *C.* *perfringens* RNA isolation, RT-PCR and RT-qPCR analysis

For obtaining RNA from TY cultures, wild-type ATCC3624, the *eutV* null mutant, and complementing strains were grown for 4 h at 37 °C. A 1-mL aliquot of each culture was removed for optical density measurement at 600 nm (OD_600_) using a Bio-Rad Smart Spectrophotometer. The equivalent OD_600_ value for each culture was then pelleted to prepare RNA.

For obtaining *C. perfringens* RNA from infected C2C12 cell cultures, the differentiated C2C12 cells in 10 cm cell culture dishes were washed twice with Hanks’ Balanced Salt Solution with calcium and magnesium but without phenol red (HBSS buffer, Corning). A 4 ml aliquot of HBSS buffer was then added to those washed cells. A 1.5 ml aliquot of overnight *C. perfringens* TY culture was also washed twice with HBSS buffer and then resuspended in 1 ml of HBSS buffer. A 200 µl aliquot of those washed *C. perfringens* cells was added to a tissue culture dish containing 4 ml of HBSS with or without differentiated C2C12 cells. Those dishes were then placed into a BD GasPak^TM^ EZ pouch (Fisher Scientific) and cultured anaerobically at 37 °C for 2 h. The number of colony forming units (CFU) in those cultures was determined by serial dilutions with PBS, followed by plating onto BHI agar. The remainder of the *C. perfringens* in each culture was pelleted by centrifugation and stored at −80 °Ç. Those pelleted *C. perfringens* cells were used to isolate RNA.

RNA was extracted using saturated phenol and purified by TRIzol and chloroform (Life Technologies and Sigma), as previously described [43]. Isolated RNA was first quantified by measuring absorbance at 260 nm. Before use in RT-PCR or RT-qRCR experiments, an aliquot (50 ng) of every isolated RNA was subjected to PCR (no RT) for *recA* to confirm that the preparation was not contaminated with DNA.

To perform RT-PCR or RT-qPCR, an aliquot (100 ng) of purified RNA was then used for first strand cDNA synthesis. The Thermo Scientific Maxima First Strand cDNA synthesis Kit was used. Reaction mixtures were incubated in a thermal cycler for 10 min at 25 °C, 30 min at 50 °C and 5 min at 85 °C to allow cDNA synthesis. For both RT-PCR and RT-qRCR, *recA* was amplified from samples and used as a loading control. RT-PCR used 10–25 ng of cDNA and RT-qPCR used 5 ng of cDNA, as previously described [41].

### Ethanolamine effects on toxin production

A 0.5% concentration of ethanolamine hydrochloride (EA, Thermo Scientific) was added to HBSS to assess EA effects on toxin production by ATCC3624. For this purpose, a 1.5 ml aliquot of an overnight ATCC3624 TY culture was washed twice with HBSS buffer and then resuspended in 1.0 ml of HBSS. A 200 µl aliquot of those washed ATCC3624 cells was added to 4 ml of HBSS or 4 ml of HBSS supplemented with 0.5% EA placed into 10 cm cell culture dishes. Those dishes were placed into a BD GasPak^TM^ EZ pouch and incubated anaerobically at 37 °C for 1 h. Bacterial pellets were collected to obtain RNA, which was used for RT qPCR to assess *eutV*, *plc,* and *pfoA* expression.

### Measurement of *C.* *perfringens* growth in TY medium or in Transwells containing HBSS with or without differentiated C2C12 cells

For analysis of *C. perfringens* growth in TY, 0.2-mL aliquots from overnight FTG cultures of the wild type, null mutants, or complementing strain were inoculated into 10 mL of TY medium. The cultures were then incubated at 37 °C; a 1-mL aliquot of culture was then removed to measure OD_600_ at 0, 2, 4, 6, 8, and 24 h culture times.

To measure *C. perfringens* growth in the presence of differentiated C2C12 cells, 12-well Transwell plates with 0.4 µm polycarbonate membranes were used (Corning). Differentiated C2C12 cells were cultured in the lower chamber. A 1.5-ml aliquot of HBSS buffer was added to those cells after two washes with HBSS buffer. A 0.6-ml aliquot of HBSS buffer with 2.5% Oxyrase for broth (Oxyrase) was added into the upper insert chamber of each Transwell. Those Transwell plates were then preincubated for 30 min at 37 °C to allow for Oxyrase to create anaerobic conditions. An ~30 µl aliquot of an overnight TY culture of each *C. perfringens* strain was then added into the upper chamber to obtain an OD_600_ equal to 0.2. At the same time, another set of Transwells was prepared without any C2C12 cells in the lower chamber. At 2, 3, 4, or 5 h after inoculation of Transwells with wild-type ATCC3624 or the *plc/pfoA* double null mutant, the OD_600_ was measured to construct a growth curve. Data for constructing growth curves were adjusted to reflect specific host cell effects on *C. perfringens* growth by calculating: OD_600_ when differentiated C2C12 cells were present in the lower chamber minus OD_600_ when differentiated C2C12 cells were absent from the lower chamber. The same method was used to measure growth of the *plc* null mutant and the *pfoA* null mutant, and their complementing strains, except that this experiment was only performed after 3 and 4 h, with the OD_600_ adjusted as described above.

### Measurement of *C. perfringens*-induced C2C12 cell cytotoxicity

Differentiated C2C12 cells cultured in 6-well plates with 1 ml of HBSS buffer were treated with a 50 µl aliquot of washed ATCC3624 cells or cells of the isogenic *eutV* null mutant or complementing strain. Treatment of differentiated C2C12 cells with HBSS buffer alone served as a negative control, while treatment of differentiated C2C12 cells with 1% Triton-X100 served as a positive control. All cultures were incubated in a GasPak pouch for 2 h at 37°C. Following the treatment, the supernatant was removed for cytotoxicity detection using an LDH-release cytotoxicity detection kit (Roche).

For determining C2C12 cell cytotoxicity in Transwell cultures, individual Transwells were pre-pared and infected as described above for the growth experiments. Supernatant in each lower chamber supernatant was removed for cytotoxicity detection after 4 h of treatment with wild-type ATCC3624, the single *plc* or *pfoA* null mutants, their complementing strains, and the double toxin null mutant strain. Cytotoxicity was then determined by measuring LDH release with a cytotoxicity detection kit (Roche).

### Western blot analyses of PLC and PFO production

TY cultures of the wild-type, null mutants or complementing strains were adjusted to an equal OD_600_. The supernatants from those cultures were then used for PLC and PFO Western blot analysis, performed as described previously [28].

### Statistical analyses

All statistical analyses were performed using Excel. All results were compared pairwise against wild-type result using Student’s T-test. Differences were considered significant when the *p* value was less than 0.05.

## Supplementary Material

Supplemental Material

## Data Availability

The data generated during the study are available at repository Figshare. The link is here: https://figshare.com/authors/ Jihong_Li/18932734
