# Supplementary figures and images for: The presence of differentiated C2C12 muscle cells enhances toxin production and growth by Clostridium perfringens type A strain ATCC3624
Source: Virulence. 2024 Aug 27;15(1):2388219. doi: 10.1080/21505594.2024.2388219 (PMC11364075; doi:10.1080/21505594.2024.2388219)

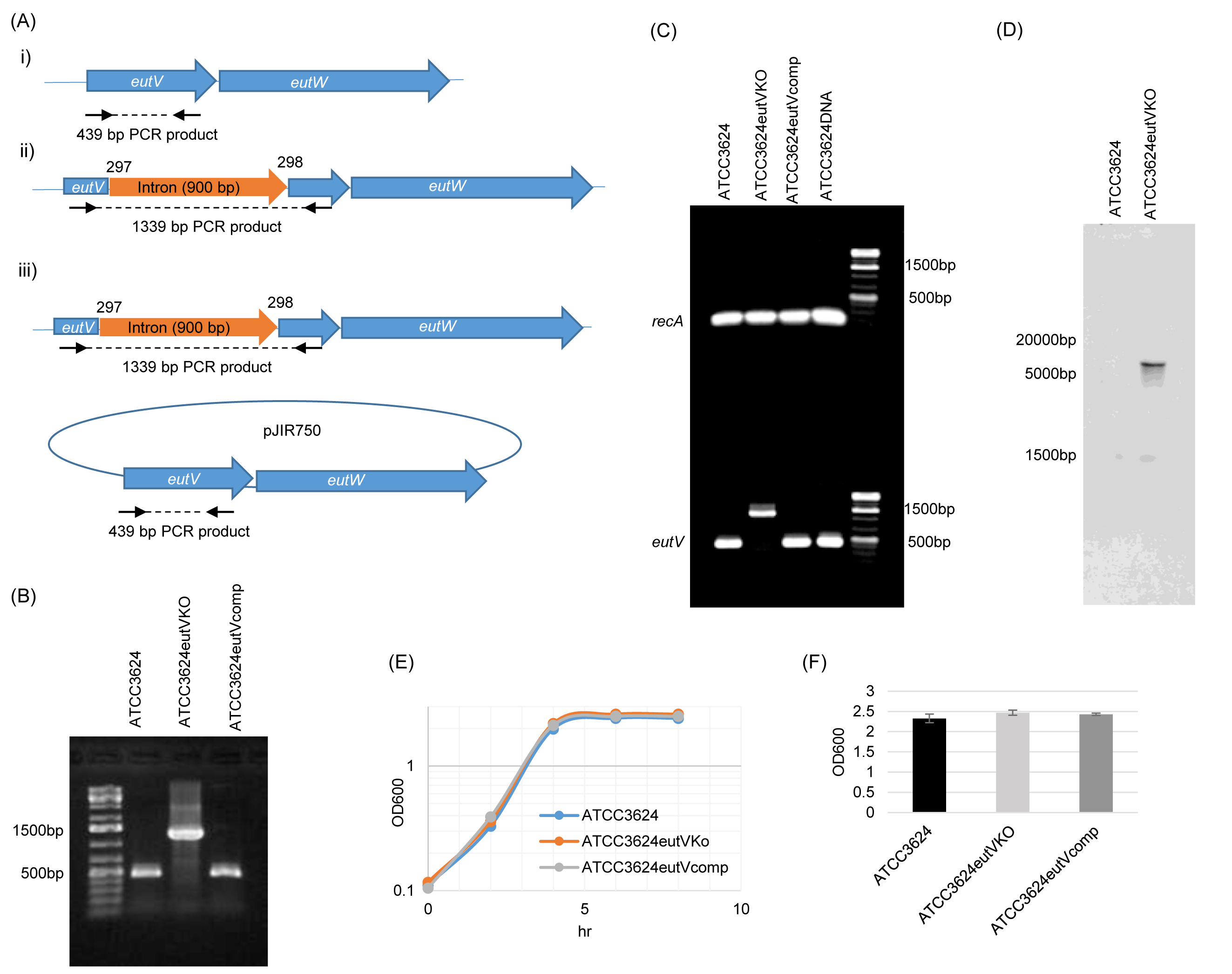

Supplement: Supplemental Material [file KVIR_A_2388219_SM2002.zip › supplement/SFig1.tif]

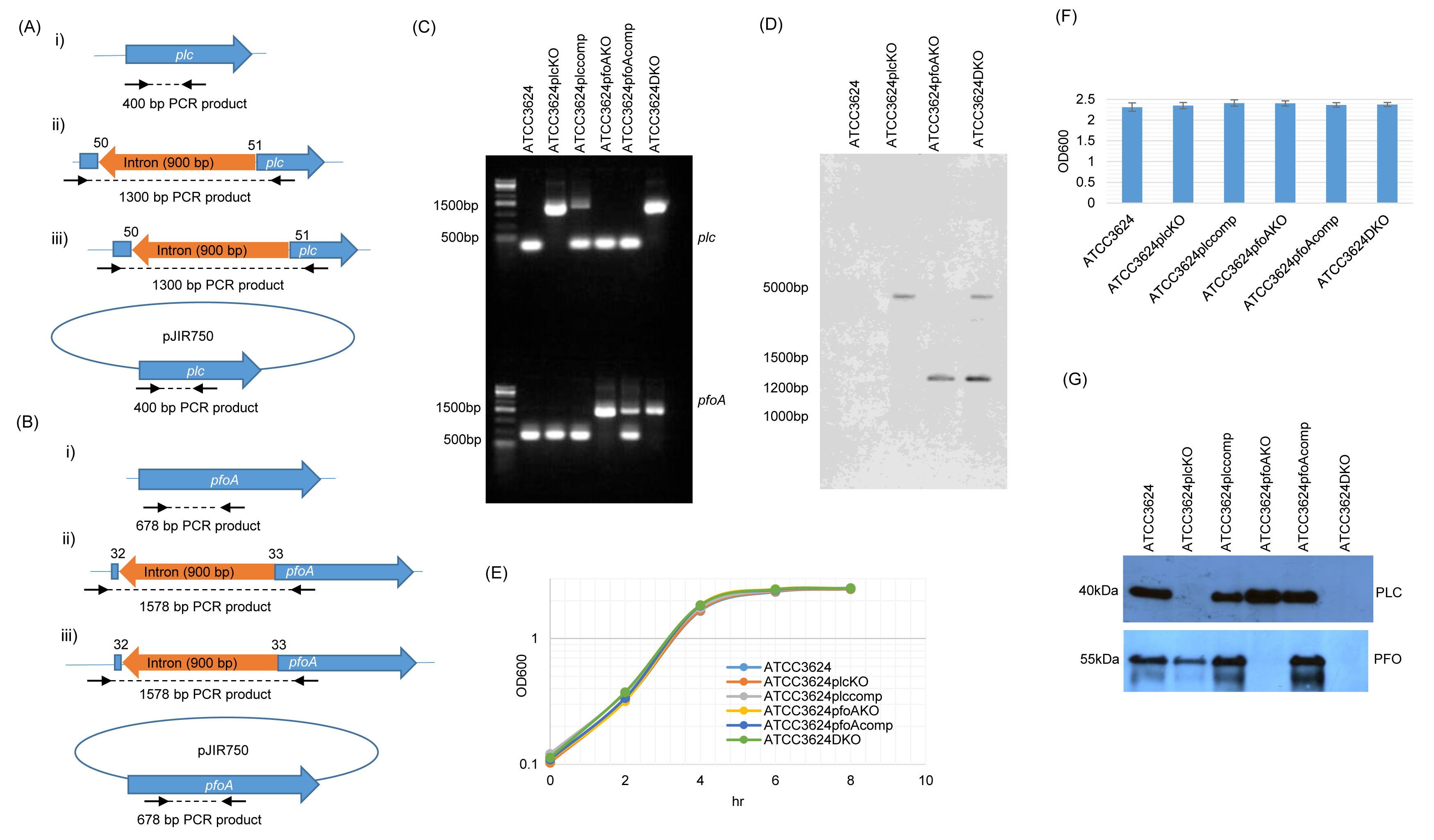

Supplement: Supplemental Material [file KVIR_A_2388219_SM2002.zip › supplement/SFig2.tif]
